# First genomic insights into the *Mandevilla* genus

**DOI:** 10.3389/fpls.2022.983879

**Published:** 2022-08-16

**Authors:** Fabio Palumbo, Samela Draga, Francesco Scariolo, Giovanni Gabelli, Gio Batta Sacilotto, Marco Gazzola, Gianni Barcaccia

**Affiliations:** ^1^Department of Agronomy, Food, Natural Resources, Animals and the Environment, University of Padova, Padua, Italy; ^2^Gruppo Padana Ortofloricoltura S.S., Treviso, Italy

**Keywords:** genome assembly, Apocynaceae, SSR, DNA superbarcoding, flow cytometry, monoterpene indole alkaloids, cpDNA, *Mandevilla sanderi*

## Abstract

*Mandevilla* (Apocynaceae) is a greatly appreciated genus in the world ornamental market. In this study, we attempted to address the poor genetic knowledge and the huge taxonomic gaps existing in this genus by analyzing a collection of 55 accessions. After cytometrically determining the triploid genome size (1,512.64 Mb) of a reference sample (variety “Mandevilla 2001”), the plastidial genome (cpDNA, 0.18 Mb) and a draft of the nuclear genome (nuDNA, 207 Mb) were assembled. While cpDNA was effective in reconstructing the phylogenesis of the Apocynaceae family based on a DNA superbarcoding approach, the nuDNA assembly length was found to be only 41% of the haploid genome size (506 Mb, predicted based on the K-mer frequency distribution). Its annotation enabled the prediction of 37,811 amino acid sequences, of which 10,562 resulted full length proteins. Among them, we identified nine proteins whose orthologs (in *Catharanthus roseus*) are involved in the biosynthesis of monoterpene indole alkaloids (MIAs), including catharanthine, tabersonine, and vincadifformine. The nuclear genome draft was also useful to develop a highly informative (average polymorphism information content, PIC = 0.62) set of 23 simple sequence repeat (SSR) markers that was validated on the Mandevilla collection. These results were integrated with cytometric measurements, nuclear ITS1 haplotyping and chloroplast DNA barcoding analyses to assess the origin, divergence and relationships existing among the 55 accessions object of the study. As expected, based on the scarce information available in the literature, the scenario was extremely intricate. A reasonable hypothesis is that most of the accessions represent interspecific hybrids sharing the same species as maternal parent (i.e., *Mandevilla sanderi*).

## Introduction

*Mandevilla* Lindl. (in honor of the diplomat Henry Mandeville) is a genus of plants greatly appreciated for their pink, white or red flowers, whose blooming time usually fluctuates from late spring to autumn. It is native to Middle and South America (Eltlbany et al., 2012) and belongs to the Apocynaceae family, along with more than 4,600 species (Bhadane et al., 2018). Several Apocynaceae family members are highly reputed in traditional medicine and have been extensively investigated for a wide range of curative properties, including antioxidant (Naidoo et al., 2021), anti-inflammatory (Bhadane et al., 2018), anticancer (Wen et al., 2016), and antimicrobial (Anand et al., 2020) activities. Mandevilla instead represents a leading product of the world ornamental scenario, as demonstrated by the growing number of new varieties that literally flood the market every year (Oder et al., 2016). Despite this, very few studies have been accomplished in this species, and many biological questions remain unsolved. Starting from the genus name, the debate about the use of *Dipladenia* (meaning “with two glands”) as a synonym of *Mandevilla* is still open. A few years after the first classification of the *Mandevilla* genus (1840), Alphonse de Candolle published in Vol. VIII of *Prodromus Systematis Naturalis Regni Vegetabilis* a revision of Apocynaceae systematics, establishing a new genus called *Dipladenia*, different from the sister genus *Mandevilla* (de Candolle, 1844). Almost 100 years later, a second classification (Woodson, 1933) revolutionized the genus organization: *Dipladenia* spp., along with seven other genera, all of which were incorporated in the *Mandevilla* genus. Although Dipladenia is currently generally considered a historical synonym of Mandevilla, a recent study demonstrated that the Dipladenia group clustered apart from the Mandevilla group (Oder et al., 2016). Moreover, some breeding companies are still tied to tradition, and they continue selling their products, clearly distinguishing Mandevilla from Dipladenia (Flowers, 2021). In fact, although from a botanical point of view this distinction is considered inconsistent, from a commercial point of view, the term Mandevilla is sometimes still used to indicate vigorous climbing varieties with large leaves, while Dipladenia is used for more compact and bushy varieties with small leaves.

In addition to these intergenus disputes, intragenus classification has also faced significant complications. The number of species included in the *Mandevilla* genus increased from 108 [from a first systematic classification, in 1933 (Woodson 1933)] to 179 [as reported by the Royal Botanic Gardens, Kew (Royal Botanical Garden Kew, 2021a)], but thus far, only 50 have been molecularly characterized and registered in BOLD systems and NCBI. Except for Simões et al. (2006), who analyzed 47 different *Mandevilla* species, exhaustive and elucidating DNA barcoding-based studies are missing in this genus. What makes this genus challenging for taxonomists is the remarkable morphological variation observed among and within species due to a continuous process of adaptation to a plethora of different geographical environments (Simões et al., 2006). Another level of complexity is given by the fact that most of the genotypes available are interspecific hybrids, characterized by complex genealogies (Oder et al., 2016) and possibly even by different levels of ploidy or by unbalanced numbers of chromosomes. Additionally, Mandevilla is an understudied species: no information about DNA content (*c*-value) and ploidy levels is available in the scientific literature.

Taxonomic classification and phylogenetic relationships are not the only lacking aspects; molecular markers, with one exception (Oder et al., 2016), have never been exploited in these species, suggesting that despite the huge turnover, breeding schemes are still planned almost exclusively on a phenotypic basis. Historically, marker-assisted breeding in ornamental species has been delayed compared to crop species for several reasons, such as reduced economic importance, genome complexity (i.e., ornamental plants have genomes that are usually larger and polyploid) and methods of propagation (i.e., ornamental plants are mainly vegetatively propagated). More recently, the collapse of sequencing costs has made next-generation sequencing platforms accessible also to non-crop species, including ornamental plants. This contributed to the development of codominant molecular markers [i.e., simple sequence repeat (SSR) and SNP] in species such rose (Zhang et al., 2006), lilium (Lee et al., 2011), tulip (Pourkhaloee et al., 2018), or chrysanthemum (Jo et al., 2015).

To take a first step in this direction, we tried to address some of the main deficits existing in this genus from both a cytometric and genomic point of view. In the first case, we first estimated the size of the genome and the DNA content of some samples commercially available on the market. In the second case, we developed and annotated a first draft of the genome with the identification of thousands of genes and markers useful for marker-assisted breeding. Notably, we assembled plastid DNA and assessed its effectiveness in taxonomy classification through DNA superbarcoding analysis.

## Materials and methods

### Plant materials

Fifty-five samples from the *Mandevilla* genus were collected as representatives of the current market. With few exceptions, no information was available about any possible origin, relatedness, or ploidy level of the accessions. Moreover, many, if not all, accessions could be interspecific hybrids. Due to the economic interest behind this species, breeding companies often contain all this information. In light of these considerations, this study always refers to the genus *Mandevilla* without specifying any species.

### Genome size estimate through flow citometry

The genome size of the “Mandevilla 2001” sample, subsequently used for genome sequencing, was determined through flow cytometry of propidium iodide (PI)-stained nuclei, following the procedure described by the CyStain PI Absolute P protocol (Sysmex Partec, Görlitz, Germany). One hundred milligrams of fresh leaf tissue was chopped with a razor blade along with 0.5 ml of Nuclei Extraction Buffer (Sysmex Partec), incubated for 45 min at room temperature and filtered using 30 μm CellTrics (Sysmex Partec). Two milliliters of staining solution (1982 μl of Staining Buffer, 12 μl of PI and 6 μl of RNAse A 3.3 ng/μl) was then added to each filtered sample, and the resulting solution was placed on ice in the dark for 45 min. Analyses were run by setting the following parameters: Nd-YAG green laser: λ = 532 nm; 30 mW, flow rate of 4 μl/s. *Raphanus sativus, Glycine max*, and *Solanum lycopersicum* seeds with known 2C DNA content were kindly provided by Prof. Dolezel,^1^ adopted as reference standards, and their relative fluorescence was used to estimate the genome size of the 2001 sample. Fluorescence histograms were evaluated using FCS Express 5 Flow software (Sysmex Partec), and *c*-values were inferred by comparing the sample and standard at G0/G1 peak positions.

### DNA extraction, library preparation, and sequencing

Due to its historical and commercial relevance, the “Mandevilla 2001” variety was selected for whole genome sequencing analysis. Leaves were collected, snap-frozen in liquid nitrogen upon harvesting and stored at –80°C until further processing. Genomic DNA was isolated using the cetyltrimethylammonium bromide (CTAB) protocol (Doyle and Doyle, 1987). gDNA integrity was first evaluated through an electrophoretic run (0.8% agarose/1× TAE gel containing 1× Sybr Safe DNA stain; Life Technologies, Carlsbad, CA, United States) and an Agilent Bioanalyzer 2100 (Agilent Technologies, Santa Clara, CA, United States). Concentration and purity (260/280-nm and 260/230-nm absorbance ratios) were spectrophotometrically assessed thorough a NanoDrop 2000c spectrophotometer (Thermo Scientific, Waltham, MA, United States).

Library preparation was accomplished using ∼500 ng of gDNA and the Illumina Nextera DNA Flex library preparation kit (Illumina, Inc., San Diego, CA, United States) following the protocol provided by the company. The library was sequenced on an Illumina NovaSeq system using paired-end, 100-bp-read chemistry and with an average insert size of 550 bp.

### Plastidial genome assembly and phylogenetic analysis

NOVOPlasty 4.2 (Dierckxsens et al., 2017) was used to assemble the chloroplast genome, adopting the cpDNA of *Rhazya stricta* (NC_024292.1, from NCBI) as the reference sequence and the *rbc*L gene of *M. sanderi* (X91764.1 from NCBI) as seed input. *R. stricta* was selected as the phylogenetically closest species to *Mandevilla* with both organelle genomes (i.e. mtDNA and cpDNA) available. The following parameters were also specified: automatic insert size detection, a genome size range from 120,000 to 180,000 (based on an estimate derived from *Rhazya stricta)*, and a K-mer value of 33. The chloroplast genome was annotated using GeSeq (Tillich et al., 2017) using *Rhazya stricta* as a reference. OGDRAW (Greiner et al., 2019) was finally employed for the graphical visualization of the circular DNA. To investigate the systematic relationships existing between the *Mandevilla* genus and the rest of the Apocynaceae family, a DNA superbarcoding-based analysis was performed by aligning the newly assembled cpDNA with the 33 plastid genomes available in NCBI (U.S. National Library of Medicine, 2021) for the abovementioned family (Supplementary Table 1). The cpDNA of *Gentiana officinalis* was selected as the outgroup since its family (Gentianaceae) is from the same order (Gentianales) of Apocynaceae. The 35 plastomes were aligned using MAFFT v7.450 (Katoh and Standley, 2013) under default parameters, while Geneious prime (Biomatters, Inc., San Diego, CA, United States) was used to reconstruct the phylogenetic relationships based on the neighbor-joining (NJ) method with 1000 bootstrap replicates.

### Nuclear genome assembly and annotation

For nuclear genome assembly, raw sequences were first processed using fastp software (Chen et al., 2018) to remove the adapter sequences and to trim low-quality bases using the following parameters: qualified_quality_phred 20, unqualified_percent_limit 30, average_qual 25, low_complexity_filter = True, complexity_threshold 30, length_required 40. The filtered reads were then assembled using MaSuRCA (Zimin et al., 2013) under the following conditions: extend_jump_reads = 0; graph_kmer_size = auto; use_linking_mates = 1; use_grid = 0; lhe_coverage = 25; mega_reads_one_pass = 1; ca_parameters = cgwerrorrate = 0.15; close_gaps = 1; jf_size = 60000000000. The same software was also used to predict a k-mer-based haploid genome size estimate by evaluating *K*-values ranging from 19 to 31.

According to the estimated haploid genome size, raw data were used to compute the sequencing depth by means of the Lander/Waterman equation:

C=LN/G

where C is the average coverage, L is read length, N is the number of reads, and G is the haploid genome length.

Assembly statistics were calculated using the NGS QC Toolkit v.2.3.3 (Patel and Jain, 2012). A BUSCO (Benchmarking Universal Single-Copy Orthologs) analysis (Simão et al., 2015), based on 2,326 single-copy orthologs included in the eudicots_odb10 database (Afgan et al., 2018), was performed to provide a quantitative assessment of the completeness in terms of the expected gene content of the genome assembly.

The gene prediction of the newly assembled genome was performed by screening those contigs whose length was higher than the 75th percentile (> 2,204 bp). Augustus v.3.4.0 (Stanke et al., 2008) was firstly trained at default parameters using the genome of *Coffea arabica* (Rubiaceae family, Gentianales order, GCF_003713225.1), being this species the phylogenetically closest one to the *Mandevilla* genus with a well-assembled and annotated genome. The resulting output was used to guide the prediction of CDSs and amino acid sequences in the selected contigs of Mandevilla. Gene annotation was then performed against the proteome of *Coffea Arabica* (GCF_003713225.1, 67,222 proteins) A BLASTp-based approach (BLAST+ v.2.3.0) was finally employed by using the predicted amino acid sequences as a query to interrogate the coffee proteome.

Since GO annotation was not available for *C. Arabica*, GO enrichment analysis was conducted by aligning (BLASTp, BLAST+ v.2.3.0) the Mandevilla proteome against the *Coffea canephora* (Rubiaceae family, Gentianales order) proteome (GCA_900059795.1). ShinyGO v.0.741 (Ge and Jung, 2018) was used to enrich those Mandevilla proteins with qcov > 95% (qcov is the ratio of length of the match by length of the query sequence).

Finally, as monoterpene indole alkaloids (MIAs) are a large group of plant compounds with enormous pharmaceutical potential that have been isolated mainly from species belonging to the Apocynaceae family (Balsevich, 1988), we tried to decipher whether Mandevilla has the orthologous enzymes necessary to produce these molecules. At this aim, 10 amino acid sequences associated with enzymes involved in the biosynthesis of the three main MIAs [i.e., catharanthine, tabersonine and vincadifformine (Qu et al., 2018a,^2019^)] in *Catharanthus roseus* (Apocynaceae) were retrieved from NCBI and used as queries in a BLASTp-based approach (*E*-value ≤ 1e-40) against the predicted amino acid sequences of Mandevilla.

### Triallelic loci prediction and simple sequence repeat identification

The filtered raw reads used for the assembly were re-aligned against the genome by means of BWA-MEM v0.7.17 software (Li, 2013). By postulating a triploid condition, a variant calling analysis was performed by means of the HaplotypeCaller function in GATK v. 4.1.4.0 software (McKenna et al., 2010; Ryan et al., 2018), using as inputs the FASTA file of the genome and the alignment file produced with BWA-MEM. The resulting VCF output was filtered, excluding the variants where genotypes had no reference alleles and the variants with coverage lower than 30 or higher than 400.

The filtered variants were annotated through the geneanno function in ANNOVAR software [16/07/2017 release (Wang et al., 2010)], at default parameters. The threshold discerning up- and downstream regions from intergenic regions was kept at 1 kbp. For an appraisal of the total of bases involved in splicing events, 12 bp per introns were considered.

Microsatellite regions (or SSRs) were searched through the MIcroSAtellite (MISA) Identification Tool Perl script (Thiel et al., 2003), screening the genome for mono-, tri-, tetra-, penta-, and hexanucleotide repeat motifs with minimum repeat numbers of 20, 10, 7, 5, 5, and 5, respectively. The maximum number of nucleotides interrupting two SSR regions in a compound microsatellite was set at 200 bp, and the space between imperfect SSR stretches was set at 5 bp. Finally, a total of 100 primer pairs (Supplementary Table 2) were randomly designed using Geneious Prime 2020 software (Biomatters Ltd.), according to the following considerations: (i) dinucleotide or trinucleotide repeat motif; (ii) length of the repeat motif ≥ 20 times (i.e., ten dinucleotide repetitions or seven trinucleotide repetitions); (iii) melting temperature (T_m_) always between 55 and 57°C to facilitate their use in multiplex reactions. For this latter reason, the 5’ end of each forward primer was tagged with an oligonucleotide tail (M13, PAN1, PAN2, or PAN3) to be employed in PCRs in combination with a complementary fluorophore-labeled (6-FAM, VIC, NED, and PET were used as fluorophores) oligonucleotide. This three-primer-based strategy is a modified version of what was reported by Schuelke (2000) and originally described in Palumbo et al. (2017).

The amplification efficiency and the polymorphic rate of the 100 SSR primer pairs were preliminarily tested in single and multiple reactions on 4 samples (including 2001), selected on the basis of marked phenotypic diversity and whose DNA was isolated using the DNeasy Plant Pro Kit (Qiagen, Hilden, Germany). The 23 SSR marker loci that amplified efficiently, generating unambiguous and polymorphic profiles (Table 1) were then organized in 4 multiplex and validated on 55 samples belonging to a Mandevilla collection whose gDNA was extracted using the DNeasy Plant Pro Kit (Qiagen).

**TABLE 1:** Information on the 23 microsatellite markers validated using a collection of 55 Mandevilla samples.

SSR	Marker Size	Primer F	Primer R	Mean T_m_ (°C)	Anchor	Multiplex
SSR_02	327	ATTTGTTTGCAACCTCCATG	CCGCAACTCAAACTCAAATT	55	PAN1	1
SSR_12	153	TGAAATAAAGGGTTAGGGCA	TCACTAATCCAGACAATCACA	54.2	PAN3	1
SSR_30	233	CAACACCTATACCTCACACC	GAGTTTGTAGTCTCCAACCTT	55.2	M13	1
SSR_34	202	TCTCCAATTAGCAGTACAAGG	TTAGACAGGGAGAGAGACAG	55	PAN1	1
SSR_41	171	GCCTCTCAAGTCATTAGGTG	AGGGTACTAAGGATGGTCTAA	55.5	PAN2	1
SSR_47	157	TGCTGCATTAATCACCTACA	GGCAGAAGAAGATTTGTCCA	54.6	M13	1
SSR_28	415	GAGATCAATGAGGATGGGAC	CACTTACAGTTTCAGGTCCT	54.6	M13	2
SSR_40	300	TGGACGAACTTGATACTACG	TGTTGAAAATCCCAGTCCAA	54.7	PAN1	2
SSR_50	138	CATTCAGCACACAGTTCTTC	AGTCATCGTTGTGAAATGGA	54.9	M13	2
SSR_15	223	TGAGGCACATACCATAGAGA	AATTTCTTGTCGTGGGCTAT	55	PAN3	2
SSR_48	187	CCGTGCCTCCTATGATTTAC	CTGACCATGCAATTACTCCT	55.1	M13	2
SSR_60	336	CCCTAGAGACCTTTTCATCC	CGAGTGTCTTCAAGCCATTA	54.5	M13	3
SSR_59	163	ATTCAGCACACAGTTCTTCT	GTCTATGACGGAGAGAAACC	54.9	M13	3
SSR_67	115	TACTAATTCGTCGTTTGGCT	CTTTTAGGTCATTTGGTCCAA	54	PAN1	3
SSR_55	185	TTTCAGCATAGGTTCGACAA	AAAGCCTGAATCTCCTCTTG	55	PAN2	3
SSR_76	272	AATAAACAGCCCAGTCTCAA	TTCTTCAATTTGCAGCCTTT	54.2	PAN3	3
SSR_89	424	AAACTGGGACCATACACATC	TTGACGTAACTGTTTGACCA	55	PAN3	3
SSR_74	253	GACGGATGCTCTTAATTCCT	GTGTACAGATCCCTACTTCC	54.3	M13	4
SSR_64	206	GGCACCTGTTAATATCAGTG	GATGGATGTAGAGGATGGTG	53.8	M13	4
SSR_70	345	TATTGAGGTTTGGCTTTCGA	CATTAACACCCCTCTTGTCA	55	PAN1	4
SSR_80	421	CTTTGGATTTGAAAGCGGAA	CAAAGGTATGTCTCTGGGTC	54.8	PAN2	4
SSR_61	413	ACAAAGCTTCTCCATCTCAG	GGGTGACTTTCCTGCTAATT	55.1	PAN3	4
SSR_95	139	ATTTTCCGTGAATCCAGATCT	TGAGAAGGGGTTGTTGTTG	55	PAN3	4

PCRs were performed in a total volume of 20 μl containing approximately 20 ng of gDNA template, 1× Platinum Multiplex PCR Master Mix (Applied Biosystems, Carlsbad, CA, United States), GC enhancer 10% (Applied Biosystems), 0.05 μM tailed forward primer (Invitrogen, Carlsbad, CA, United States), 0.1 μM reverse primer (Invitrogen), 0.23 μM universal primer (Invitrogen) and sterile water to volume. A 9600 Thermal Cycler (Applied Biosystems) with 96-well plates was used for PCRs setting the following thermal conditions: 5 min at 95°C, followed by three cycles at 95°C for 30 s and at 54°C for 45 s, which decreased by 1°C with each cycle, and at 72°C for 45 s; then 37 cycles at 95°C for 30 s, at 51°C for 45 s, and at 72°C for 45 s. Reactions were terminated with a final extension of 30 min at 60°C.

PCR products were first run by means of agarose gel electrophoresis (agarose 2% agarose/1× TAE gel containing 1× Sybr Safe DNA stain; Life Technologies) and visualized on an Uvidoc HD6 transilluminator (Uvitec, Cambridge, United Kingdom) equipped with a digital camera. They were then dried at 65°C for 1 h and subjected to capillary electrophoresis (ABI PRISM 3130xl Genetic Analyzer, Thermo Fisher) with LIZ500 (Applied Biosystems) adopted as molecular weight standard.

The allele size of each SSR locus was determined through Peak Scanner Software 1.0 (Applied Biosystems), while the observed (Ho) and expected (He) homozygosity, the Shannon index (I) of phenotypic diversity, and the number of observed (Na) and effective (Ne) alleles were calculated with POPGENE v.1.32 software (Yeh et al., 1997). The polymorphism information content (PIC) and thus the informativeness of each microsatellite were assessed with the Excel Microsatellite Toolkit (Park, 2001) as:

$$\text{PIC}=1-\sum_{\text{i}=1}^{\text{n}}{\text{p}}_{\text{i}}^{2}-\sum_{\text{i}=1}^{n-1}\sum_{\text{j}=\text{i}+1}^{\text{n}}2\,p_{\text{i}}^{2}\,{\text{p}}_{\text{j}}^{2}$$

Pairwise genetic similarity estimates were calculated in all possible comparisons among the 55 Mandevilla samples using NTSYS v.2.2 software (Rohlf, 1988) and by applying the Rohlf coefficient. The resulting triangular matrix was used, in turn, to build a UPGMA dendrogram. Finally, an attempt to reconstruct the genetic structure of the core collection was accomplished by modeling a Bayesian clustering algorithm available in STRUCTURE v.2.2 (Falush et al., 2003). Ten replicate simulations were conducted for each value of K (range: 2–30) as suggested by Evanno et al. (2005), while a burn-in of 2⋅10^5^ and a final run of 10^6^ Markov chain Monte Carlo (MCMC) steps were set. STRUCTURE HARVESTER (Earl and VonHoldt, 2012) was run to estimate the most likely *K* value, while estimates of membership were plotted as histograms using an Excel spreadsheet.

### Ploidy level estimates through flow citometry

The ploidy level of the 55 accessions (each analyzed in three biological replicates) was studied through flow cytometry (CyFlow Ploidy Analyzer) of 4’,6-diamidino-2-phenylindole (DAPI)-stained nuclei following the procedure described by the CyStain UV Precise P protocol (Sysmex Partec). One hundred milligrams of fresh leaf tissue taken from each of the three biological replicates were co-chopped with a razor blade in a Petri dish with 0.5 ml of Nuclei Extraction Buffer (Sysmex Partec) and incubated for 45 min at room temperature. After filtering (30 μm of CellTrics^®^, Sysmex Partec), 2 ml of staining buffer was added to each sample and incubated for 60 s before analysis (Nd-YAG green laser: λ = 532 nm; 30 mW, flow rate of 4 μl/s). Fluorescence histograms were evaluated using FCS Express 5 Flow software (Sysmex Partec), and ploidy levels were inferred by comparing each sample with the ploidy level of the 2001 sample.

### Assessing the phylogenetic origin of the mandevilla germplasm

The DNA of the 55 samples previously genotyped with SSR markers and cytometrically analyzed for ploidy level investigation was also analyzed at the plastidial gene encoding the ribulose bisphosphate carboxylase large chain (*rbc*L) and at nuclear internal transcribed spacer 1 (ITS1). Primer pairs adopted, along with the relative nucleotide sequences, are available in Scariolo et al. (2021). PCRs were performed in 25 μL containing 50 ng of genomic DNA as a template, 12.5 μL of MangoMix (Bioline, London, United Kingdom), 2 μL of each primer (10 mM) and sterile water to reach the final volume. A Veriti 96-Well Thermal Cycler (Applied Biosystems) was used to carry out the amplifications by setting the following parameters: 2 min at 95°C; 35 cycles at 95°C for 30 s, 55°C for 45 s, and 72 °C for 45 s; and a final extension at 72°C for 10 min. PCR products were purified with ExoSAP-IT PCR Product Cleanup Reagent (Thermo Fisher) and sequenced on an ABI 3730XL Genetic Analyzer (Applied Biosystems). Chromatograms were evaluated using Geneious Prime software,^2^ and sequences were trimmed at the 5’ and 3’ positions to remove low-quality regions. The clean *rbc*L and ITS1 sequences of each sample were examined in the BOLD system and GenBank, respectively, by means of a BLASTn search and were then concatenated and globally aligned with those of the other samples (Clustal omega algorithm, Geneious software). The resulting multiple alignment was used for the construction of a neighbor-joining (NJ) tree using the Juke-Cantor algorithm (a bootstrap analysis was conducted to measure the stability of the computed branches with 1,000 resampling replicates). Polymorphic sites were used to create a logo graph.

## Results and discussion

### Genome size estimate through flow citometry

**FIGURE 1:**
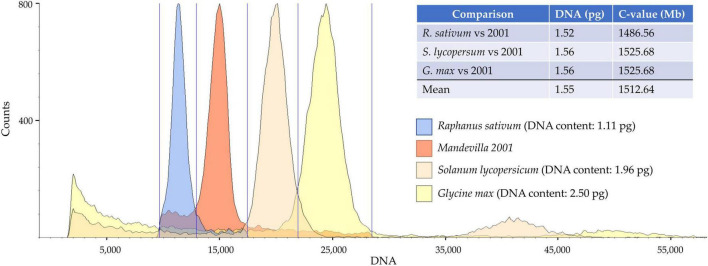
Flow cytometry analysis for genome size estimates. Each peak represents the total DNA fluorescence emission of propidium iodide (PI)-stained leaf nuclei purified (in order from left to right) from *Raphanus sativum*, Mandevilla 2001, *Solanum lycopersicum*, *Glycine max.*

Genome size data for the Apocynaceae family are quite scant: only 35 species (of approximately 4,600) have been cytometrically investigated, and the *Mandevilla* genus is not among them (Royal Botanical Garden Kew, 2021b). In this study, the genome size of “Mandevilla 2001” was successfully determined by costaining its nuclei along with those extracted from *Raphanus sativus*, *Glycine max*, and *Solanum lycopersicum*. These reference standards were chosen because the sizes of their genomes are placed in a range (from 1,085 Mb for *R. sativus* to 2,446 Mp for *G. max*) sufficiently wide to cover the putative genome size of *Mandevilla* spp. In fact, although data about the DNA content of this genus are lacking, the genome size for most of the known Apocynaceae species ranges from 490 Mb (*Apocynum androsaemifolium*) to 2,322 Mb (*Vinca difformis*). Notably, despite the usage of three references from as many families, estimates of 2001 sample genome size provided highly overlapping values, ranging from 1.52 to 1.56 pg (Figure 1). The slight difference (3.4%) observed among the measurements, according to Bennett et al. (2008) could be due to the presence of inhibitors (e.g., anthocyanin) preventing PI binding to DNA or variability in chromatin condensation across samples (Bennett et al., 2008). Overall, assuming 1 pg = 0.978 Gbp (Dolezel et al., 2003), the genome size of the Mandevilla sample oscillates between 1,486.56 and 1,525.68 Mb.

### Sequencing output, organelle, and genome assembly

**FIGURE 2:**
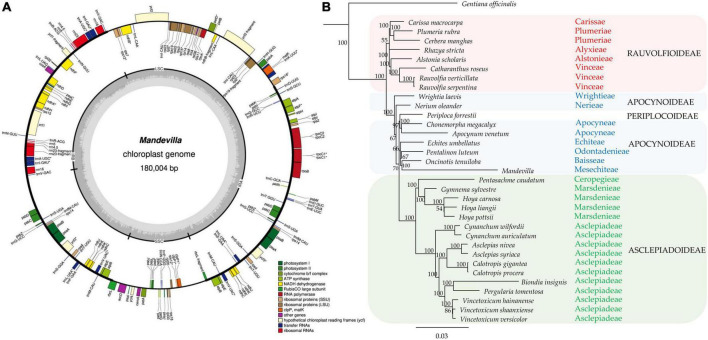
**(A)** Annotation of the Mandevilla 2001 plastidial genome. Genes marked with an asterisk (*) contain introns. **(B)** The newly assembled Mandevilla plastome was aligned using MAFFT v7.450 under default parameters with 33 plastid genomes (from the Apocynaceae family) retrieved from GenBank; Geneious prime (Biomatters, Inc., San Diego, CA, United States) was used to reconstruct the phylogenetic relationships based on the neighbor-joining (NJ) method, and 1,000 bootstrap replicates. *Gentiana officinalis* (Gentianaceae family) was used as an outgroup.

Whole-genome sequencing yielded 314,414,127 × 2 reads (∼62.8 Gb); 97.3% of them had a Phred quality score > 20, and 91.7% had a Phred quality score > Q30. After adapter removal, a quality check (Phred Quality Score > 30) and a trimming step (read length > 40 bp), 56.41 Gb were retained and used for both plastid and nuclear genome assembly. The chloroplast genome assembly (deposited in GenBank under accession ID OM489306), with a length of 180,004, resulted in the largest plastid organelle assembled from its family. In fact, the 33 cpDNA available in NCBI for the Apocynaceae family have a size ranging from 153,826 (*Periploca forrestii*) to 176,340 (*Hoya carnosa*). The chloroplast genome showed a small single copy (SSC), a large single copy (LSC), and two inverted repeats (IRA and IRB, Figure 2A). These latter have been reported, with few exceptions, in almost all land plants and are considered an ancestral feature that was lost independently in several plant families (Plunkett and Downie, 2000). Overall, the genome contained 107 different genes, including 73 protein-coding, 30 tRNA, and 4 rRNAgenes (Supplementary Table 3). Among them, 10 genes contained one intron, and 6 genes (*trn*V-UAC, *trn*I-GAU, *trn*A-UGC, *trn*L-UAA, *rpl2*, and *ycf3*) contained two introns. The newly assembled plastome was used to investigate the phylogenetic relationship between the *Mandevilla* genus and 34 other Apocynaceae species based on a DNA superbarcoding approach. From the plastome-based tree, all species were correctly grouped into their 15 relative tribes with bootstrap values usually equal to 100 (Figure 2B). Mandevilla, which is part of the Mesechiteae tribe, clustered with all tribes belonging to the Apocynoideae subfamily. Moreover, the relationships existing among the four subfamilies (Rauvolfioideae, Apocynoideae, Periplocoidae, and Asclepiadoideae) perfectly matched those reported by Livshultz et al. (2007) and by Livshultz (2010) although the analyses were conducted differently (i.e., aligning entire plastomes and single plastidial genes such as *trn*L intron, *trn*L-*trn*F spacer, *rpl16* intron, and *mat*K).

Regarding the nuclear DNA assembly, filtered and merged paired-end data were assembled into 116,244 contigs encompassing a total of 207,657,661 bp (Table 2). The genome draft was deposited in GenBank under accession ID PRJNA802340.

**TABLE 2:** Assembly statistics and BUSCO analysis results.

Assembly statistics	
N. contigs	116,244
Total length (bp)	207,657,661
N. contigs > 500 bp	86,428
Largest contig (bp)	36,523
Total length (bp; > 500 bp)	197,539,476
GC (%)	38.38
N50 > 500 bp	3,335
L50 > 500 bp	16,714
Coverage	62×

**BUSCO analysis (2,326 total BUSCO groups)**	

Complete	835 (816 single copy)
Fragmented	470

Additionally, based on the K-mer frequency distribution analysis (*K* = 25), the haploid genome size was estimated to be 506 Mb. According to the genome size estimates obtained using flow cytometry (∼1,512 Mb), it was possible to predict the triploidy of the Mandevilla sample. Moreover, comparing the assembled genome and the estimated haploid genome, 41% of the genome resulted assembled. This is in agreement with the BUSCO analysis findings: the assembly retrieved 55.3% of the conserved single-copy ortholog genes, including 35.1% complete and 20.2% partial genes (Table 2). Out of 116,244 contigs, 86,428 had a size ranging from 500 to 36,523 bp (N50 = 3335 bp; L50 = 37,125, Table 2). Finally, by applying the Lander/Waterman equation, the average coverage was found to be approximately 62×.

### Gene prediction and annotation

**FIGURE 3:**
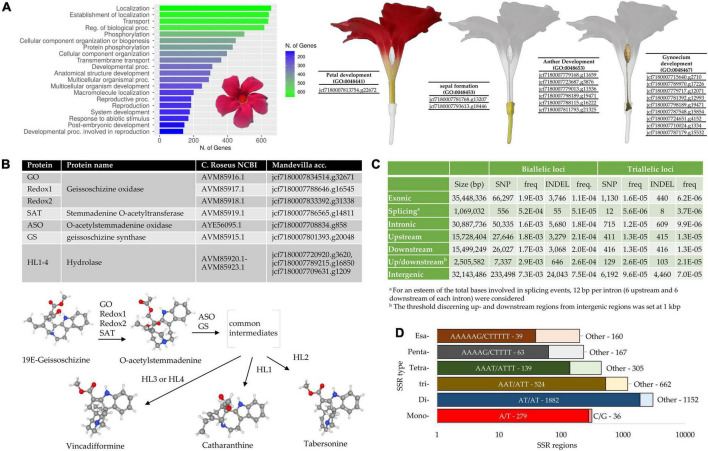
**(A)** GO enrichment analysis of the Mandevilla proteome based on the *Coffea canephora* proteome (Rubiaceae family, Gentianales order, GCA_900059795.1). The top 20 Biological Process (BP) categories (in terms of genes number) are reported. The 186 proteins involved in the “reproductive process GO:0022414” and “reproduction GO:0000001” categories were further investigated to identify amino acid sequences specifically involved in floral tissues development. **(B)** Identification of putative enzymes involved in the biosynthesis of catharanthine, tabersonine and vincadifformine. A BLASTp analysis was performed by aligning 10 amino acid sequences from *Catharanthus roseus* involved in the production of the abovementioned MIAs (monoterpene indole alkaloids) against the predicted Mandevilla proteome. In the bottom part of the panel, the last steps leading to the production of catharanthine, tabersonine and vincadifformine are illustrated according to Qu et al. (2019); **(C)** Biallelic and triallelic loci (SNP and INDEL) detected within the newly assembled genome, classified according to their location. Frequencies are also provided and they were calculated as the number of loci divided by the total length of each category (second column); **(D)** Detailed abundance of microsatellites in the newly assembled genome of Mandevilla. The most abundant motif for each SSR type is reported.

Augustus software led to the prediction of 37,811 amino acid sequences. Based on a BLASTp analysis against the *Coffea arabica* proteome, 36,204 amino acid sequences found a match against as many coffee proteins. Of these, 22,027 had *E*-values < 1e-20 (Supplementary Table 4). Noteworthy, 10,562 amino acid sequences showed a subject coverage > 95% and were thus considered full length proteins. As regards the GO enrichments analysis, the most enriched Biological Process categories in terms of absolute number of genes were “Localization,” “Establishment of localization,” “Transport,” “Regulation of biological processes,” and “phosphorylation” (Figure 3A). One hundred and eighty-six proteins resulted involved in “Reproduction” (GO:0000003) and “Reproductive process” (GO:0022414) categories and were further investigated to identify amino acid sequences involved in floral tissues development. Among them, we identified proteins specifically involved in sepal, petal, anther, and gynoecium development (Supplementary Table 4 and Figure 3A).

Among the full-length amino acid sequences identified within the newly assembled genome, we searched for sequences putatively involved in the production of MIAs. MIAs represent a large group of compounds mainly isolated from three plant families (the Loganiaceae, Apocynaceae, and Rubiaceae) and classified based on the geometric arrangement of the C9/C10 carbon skeleton in the three main configurations: Corynanthe, Aspidosperma, and Iboga types (Balsevich, 1988). The isolation in *Catharanthus roseus* (Madagascar periwinkle, Apocynaceae) of two MIAs (vinblastine and vincristine) able to block cell division is considered a milestone in cancer chemotherapy (Qu et al., 2019). Since then, several MIA-based drugs have been under consideration for different types of cancer treatment. Due to the great interest behind the production of these molecules, several studies have recently attempted to elucidate the metabolic pathway leading to the production of catharanthine (iboga MIA), tabersonine (aspidosperma MIA) and vincadifformine (aspidosperma MIA) (Qu et al., 2018a,b, 2019). The first two represent precursors of vinblastine, while the synthesis of the last compound appears to branch off prior to the formation of tabersonine (Figure 3B). Although the identification of these MIAs has been demonstrated in *C. roseus*, it has yet to be elucidated how many other Apocynaceae species can produce this class of molecules. In a recent study, 444 IMAs, including catharanthine, vincadifformine, and tabersonine, were identified in six genera belonging to this family (*Alstonia*, *Rauvolfia*, *Kopsia*, *Ervatamia*, *Tabernaemontana*, and *Rhazya*) (Mohammed et al., 2021). However, the production of MIAs in the *Mandevilla* genus has never been investigated. From the BLASTp analysis performed by aligning 10 amino acid sequences involved in the biosynthesis of catharanthine, tabersonine, and vincadifformine in *C. roseus* (Apocynaceae) against the Mandevilla proteome, we identified (*e*-values ranging from 6.47e-97 to 4.64e-171) two putative geissoschizine oxidases, a stemmadenine O-acetyltransferase, an O-acetylstemmadenine oxidase, a geissoschizine synthase and three putative hydrolases (Figure 3B and Supplementary Table 5). The latter, orthologous to HL1-HL4, could be specifically involved in the last step of biosynthesis of the three abovementioned MIA compounds. However, considering the high similarity values shared by the HL genes, we were not able to discriminate whether a specific sequence that encoded a hydrolase was involved in an IMA pathway (e.g., tabersonine) rather than another (e.g., catharanthine). qPCR analysis on target genes conducted in different tissues and at different stages of development (Palumbo et al., 2019) conjugated with liquid chromatography–mass spectrometry-based analyses will be crucial to elucidate if and possibly which MIA *Mandevilla* genus is able to produce.

### Triallelic loci prediction and simple sequence repeat screening

By aligning the raw reads against the newly assembled genome, we were able to predict 452,214 biallelic and 7,728 triallelic loci (SNPs and INDEL). By taking advantage of the gene prediction previously made using Augustus, we classified each of these loci according to its location (Figure 3C). Nearly all the biallelic loci resulted SNPs (91.0%), whereas INDEL constituted a significant amount of the triallelic loci (41.7%). The large percentage of triallelic INDEL could be partially explained by both the quality and the size of the assembled portion of the genome (41%). As expected in both SNP and INDELs the highest frequencies were registered in the intergenic regions, followed by the flanking genes regions.

Overall, the conspicuous number of triallelic loci would corroborate the triploidy of the sample used for sequencing, as initially estimated through flow cytometry measurements. Finally, those SNPs and INDELs that were predicted to be located in exonic regions were further investigated to decipher any possible effect on the resulting protein (Supplementary Table 6).

In terms of microsatellite compositions, we identified 5.408 SSRs distributed over 4,806 contigs. The total length of the SSR-containing motifs detected in the genome draft was 66,135 Mb, barely 0.032% of the assembled sequences. This value, which is ten to thirty times lower than that observed in other plant species (Palumbo et al., 2021), could depend on both the stringent parameters used to search for SSRs (e.g., minimum repeat numbers of 20, 10, and 7 for mono-, di-, and trinucleotide repeat motifs, respectively) and the type of sequencing platform used. In fact, since genome assembly relied solely on Illumina sequencing, the exclusive use of short sequences can lead to an enrichment of the coding regions and, vice versa, to an underestimation of repeated regions that, in many cases, cannot be properly assembled (unless the repeated region is shorter than the read length) (Carvalho et al., 2016). This hypothesis seemed to be confirmed by the predictions made using Augustus and ANNOVAR software (second column in Figure 3C): the exonic and intronic regions represent nearly the 50% of the total bases. The most frequent microsatellite repeats were dinucleotides (56.10%) and trinucleotides (21.93%), while the most abundant dinucleotide and trinucleotide repeat motif types were AT/AT (62.03% of all dinucleotides) and AAT/ATT (44.18% of all trinucleotides; Figure 3D). The fact that these motifs were the most abundant is fully in agreement with what was highlighted by Srivastava et al. (2019) in a recent SSR meta-analysis performed on 71 plant species.

From an initial panel of 100 SSRs (Supplementary Table 2), 23 SSRs (Table 1) were successfully employed to analyze, in multiplex, the 55-sample germplasm collection.

Descriptive statistics demonstrated a high PIC for almost all the loci, with values ranging from 0.36 (SSR_28) to 0.82 (SSR_59) and an average value of 0.62. Based on the Botstein et al. (1980) classification, the majority of SSRs (18 loci, 78%) were highly polymorphic, with PIC values always higher than 0.5. Moreover, it is worth pointing out that the overall PIC of this new set of SSR panels was demonstrated to be higher than that of the only other SSR set available for the *Mandevilla* genus (Oder et al., 2016). In this latter study, Oder et al. (2016) tested a set of 20 SSRs on 11 accessions belonging to the *Mandevilla* genus, and their PIC values oscillated from 0.08 to 0.76, and the average was 0.48 (Table 3).

**TABLE 3:** Simple sequence repeat descriptive statistics reporting marker locus name, sample size of individuals successfully amplified for each locus, number of observed alleles (n_a_), number of effective alleles (n_e_), observed heterozygosity (H_o_), expected heterozygosity (H_e_), Shannon’s information index (I), and polymorphic information content (PIC).

Marker	Sample size	n_a_	n_e_	H_o_	H_e_	I	PIC
SSR_02	53	3	2.04	0.51	0.51	0.74	0.51
SSR_12	52	4	2.98	0.88	0.67	1.18	0.66
SSR_15	45	5	3.72	0.91	0.74	1.41	0.73
SSR_28	47	3	1.55	0.30	0.36	0.60	0.36
SSR_30	45	4	1.78	0.20	0.44	0.78	0.44
SSR_34	50	7	3.88	0.76	0.75	1.59	0.74
SSR_40	46	6	3.66	0.67	0.73	1.41	0.73
SSR_41	50	10	4.18	0.70	0.77	1.69	0.76
SSR_47	55	6	1.64	0.36	0.39	0.86	0.39
SSR_48	49	5	2.11	0.43	0.53	0.95	0.53
SSR_50	51	9	4.99	0.90	0.81	1.80	0.80
SSR_55	55	4	3.32	0.84	0.71	1.27	0.70
SSR_59	55	8	4.92	0.93	0.80	1.75	0.82
SSR_60	40	3	1.81	0.08	0.45	0.68	0.45
SSR_61	53	6	2.39	0.43	0.59	1.20	0.58
SSR_64	55	4	2.15	0.60	0.54	0.87	0.53
SSR_67	54	4	1.83	0.37	0.46	0.83	0.45
SSR_70	53	6	3.59	0.66	0.73	1.50	0.72
SSR_74	52	5	3.15	0.46	0.69	1.30	0.68
SSR_76	54	6	3.13	0.67	0.69	1.38	0.68
SSR_80	53	4	2.72	0.70	0.64	1.10	0.63
SSR_89	49	6	2.04	0.53	0.52	1.01	0.51
SSR_95	54	8	4.23	0.87	0.77	1.62	0.76
Mean	51	5.48	2.95	0.60	0.62	1.20	0.62
St. dev.	4	1.93	1.06	0.24	0.14	0.37	0.14

### Investigating mandevilla germplasm by intersecting simple sequence repeat data, ploidy level, and DNA barcoding

**FIGURE 4:**
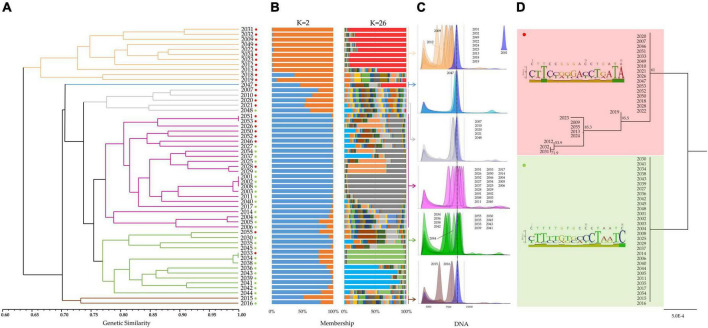
**(A)** UPGMA dendrogram based on a pairwise genetic similarity matrix (Supplementary Table 7). Six main clusters highlighted with different colors (ocher, light blue, gray, violet, green, and brown) were found within the germplasm collection of Mandevilla (*n* = 55). Red and green dots indicate the membership of each sample to one of the two clusters identified by targeted-region DNA sequencing **(D)**. **(B)** Genetic structure of the Mandevilla germplasm collection as estimated by STRUCTURE using the SSR marker dataset. Each sample is represented by a vertical histogram partitioned into *K* = 2- and *K* = 26-colored segments that represent the estimated membership (Supplementary Figure 1). The proportion of ancestry (%) is reported on the abscissa axis. **(C)** DAPI-based flow cytometry measurements of the 55 Mandevilla accessions. Each sample was analyzed by co-staining its nuclei along with those extracted from 2001 (the reference sample whose degree of ploidy was known, i.e., triploid). Samples were then grouped and analyzed according to the six clusters identified in panel **(A)**. **(D)** Neighbor-joining tree built by concatenating the ITS1 and *rbc*L sequences of each sample and aligning them through the Clustal omega algorithm. Bootstrap values are reported. For each of the two groups identified through DNA barcoding (*rbc*L) and DNA haplotyping (ITS1), in the left part of the panel, a polymorphic site-based sequence logo is reported.

The newly developed SSR set was used to investigate the level of genetic differentiation existing among the 55 accessions by calculating genetic similarity estimates for all possible pairwise comparisons. Genetic similarity (GS) values (mean = 0.72) ranged from 0.51 (2016 vs. 2009) to 1.00 (2001 vs. 2002, 2001 vs. 2008 and 2002 vs. 2008; Supplementary Table 7). Very low similarity values (such as in the case of 2016 vs. 2009) could be considered an indication that they actually belong to different species. In contrast, similarity scores close to 1.00 were often confirmed at the phenotypic level. For example, 2001, 2002, and 2008 were morphologically very similar. The common origin of these three samples is also confirmed by the fact that they were all constituted by the same breeder. Furthermore, the 2003 sample, which is known to be the result of a 2001 spot mutation, shows with the latter a degree of genetic similarity close to 100% (0.99). The GS-based UPGMA tree revealed a marked grouping of the samples into six clusters, highlighted in Figure 4A with different colors.

Microsatellite data were also used to investigate the genetic structure of the collection and all the possible ancestors. Following the procedure described by Evanno et al. (2005), a clear maximum for ΔK values was found at *K* = 2 and *K* = 26 (Supplementary Figure 1). For *K* = 2 (Figure 4B), all individuals grouping together in the UPGMA tree under the ocher cluster showed an individual membership to their founding group (orange) higher than 92% (except for 2018, 63%). In contrast, the 23 samples from the largest UPGMA cluster (violet) exhibited an average membership to the other founding group (blue) higher than 97%. All other samples were, with few exceptions (i.e., 2035, 2039, 2041, and 2045), admixed. Analyzing the same collection for *K* = 26 (Figure 4B), some subgroups emerged, both confirming the groupings identified for *K* = 2 and strengthening the common origin of some accessions. This is the case for 2001, 2002, 2003, and 2008 (gray group) or 2036, 2039, 2041, 2042, and 2043 (light blue group). Additionally, 2033, 2034, and 2038 proved to share a common ancestor (pale green group), as already demonstrated by their average genetic similarity (99%) and by the information available from the literature. An attempt to correlate this genetic evidence with the DNA content of each sample was made by taking advantage of the cytometric analyses.

The 55 Mandevilla accessions were therefore analyzed by co-staining their nuclei (DAPI) along with those extracted from 2001, as this latter was the only sample whose degree of ploidy was known (triploid). Unfortunately, considering that each accession is, in all likelihood, the result of repeated interspecific crossings, the use of 2001 as a reference to establish the degree of ploidy of the other 54 samples proved unsuccessful. In fact, the ratio between the median fluorescence intensity of each sample (MFI_s_) and the MFI of 2001 (MFI_2001_) oscillated between 0.49 and 1.14 (Supplementary Table 8). For this reason, except for those accessions whose MFI_s_/MFI_2001_ ratio was approximately 1 (and for which triploidy can be postulated), it was not possible to assign a defined ploidy value to most of the samples. For example, in the case of 2009 and 2015, with MFI_s_/MFI_2001_ ratios of approximately 0.5, it is impossible to explain a non-integer ploidy value (i.e., 1.5), and it is more likely that these samples represent species or interspecific hybrids different from those of 2001. The genus *Mandevilla* is characterized by a variable number of chromosomes [i.e., 2*n* = 16, 2*n* = 20, 2*n* = 22 (Sanso and Xifreda, 2000; Andriyanova et al., 2014)] and without karyological data, defining the number of chromosomes and chromosome pairs inherited from an interspecific hybrid is challenging. At the same time, the strong interfertility existing among the species of this genus could have favored the occurrence of dysploidy (Andriyanova et al., 2014), complicating the karyotypic scenario through gains and losses of single chromosomes or fission and/or fusion of chromosome segments (Winterfeld et al., 2020). Based on this new perspective, the effectiveness of the SSR set in analyzing all species/interspecific hybrids of the germplasm must be acknowledged. The fact that all 23 loci showed at most two alleles per locus would also support the idea that the genomic sequences used for SSR selection were derived from a diploid progenitor common to all accessions.

Although the lack of adequate references and genealogical background hindered the definition of the ploidy level, the cytometry data were still considered to highlight the extent—in terms of DNA content—of the gap existing between each sample and 2001 (Figure 4C). In this regard, it is worth highlighting how the lower values of DNA content were registered in the two sample groups (ocher and brown) that – based on the SSR-based UPGMA tree – clustered apart from 2001 (mean MFI_ocher_/MFI_2001_ = 0.77 and mean MFI_brown_/MFI_2001_ = 0.69; Supplementary Table 8). In contrast, the average MFI_violet_/MFI_2001_ ratio calculated for samples sharing the same SSR-based cluster as 2001 (violet) was close to 1 (0.99). Although it is ventured to find an explanation capable of correlating DNA content and SSR-based genetic analyses, it could be hypothesized that sharing a common genealogy (e.g., sharing a parent/ancestor) could explain both a high genetic similarity and a comparable DNA content.

To clarify the genetic relationships theorized through the SSR data and the MFI_s_/MFI_2001_ ratios, a target-DNA region sequencing analysis was conducted by means of a plastidial DNA barcoding (*rbc*L) and a nuclear DNA haplotyping (ITS1). Various coding and non-coding regions of plastid genomes and nuclear regions have been proposed, alone or in combination, for DNA barcoding studies in Apocynaceae. Among these spacers, the *trnH*-*psbA* spacer was proposed in combination with *matK* (Mahadani et al., 2013) or ITS2 (Lv et al., 2020), *rbc*L was combined with *atpB*, *rpoC1*, *trnH*-*psbA*, and ITS1/2 (Selvaraj et al., 2015), and the *trnL-F* efficiency was compared with that of *matK, rbc*L, and *trnH-psbA* (Cabelin and Alejandro, 2016). By reviewing the main DNA barcoding studies performed in this family, we found contrasting opinions on which region is the most recommended, as the type of analysis (intragenus vs. intergenera) seems to greatly influence the suitability of the barcoding region. However, several works recognized a higher efficiency in the combined use of plastidial and nuclear markers (Selvaraj et al., 2015; Mishra et al., 2017; Lv et al., 2020). For this reason, we chose both the plastidial *rbc*L region – one of the two core barcodes established by the Consortium for Barcode of Life, (CBOL; CBOL Plant Working Group, 2009) – and ITS1, a supplementary nuclear barcode candidate suggested again by the CBOL.

The obtained sequences were 632 bp (*rbc*L) and 306 bp (ITS1) long. In particular, *rbc*L was polymorphic only at position 575 (A > C), splitting the core collection into two groups of 30 and 25 accessions. Both *rbc*L sequences were used to interrogate the BOLD database, and the best match was *Mandevilla sanderi* (100% query coverage), with a slight difference in terms of identity values (100%, first sequence and 99.84% second sequence). It can be hypothesized that the maternal lineage of all the samples is likely to be the same and that the SSR panel worked in all the interspecific hybrids of the germplasm because they were (casually) selected on the portion of the maternal genome common to all. It is worth emphasizing that the smallest group (575: C) included all the samples belonging to the SSR-based ocher cluster (red dots in Figure 4A), in line with what emerged from the genetic structure analysis and from the considerations made about the MFI_s_/MFI_2001_ ratios. This would further support the hypothesis that these samples have a very distinct origin from the other clusters. ITS1, despite being shorter than *rbc*L, was polymorphic in 15 positions (15/306, Supplementary Table 9). All the ITS1 sequences, searched in GenBank through blastN, had *Mandevilla atroviolacea* as the best match with full query coverage (always 100%) but an identity score ranging from 98.52 to 95.45%. This relatively low degree of genetic identity must be interpreted considering the very limited number of ITS1 sequences available in GenBank for the *Mandevilla* genus. It is extremely likely that the species/hybrids to which the germplasm samples belong are not represented in GenBank and thus cannot be properly identified. However, by predicting – at each polymorphic position – the genotypic combinations of the two putative parents, we were able to predict the specific parental genotypes that distinguished the two groups of samples previously identified by means of *rbc*L (Supplementary Table 9).

Finally, an NJ dendrogram was built by concatenating the ITS1 and *rbc*L sequences of each sample and aligning them through the Clustal omega algorithm. Overall, samples clustered in the same two groups identified solely based on *rbc*L (Figure 4D). In addition, a further subgroup that included all the samples from the SSR-based ocher cluster (Figure 4A) was identified, again corroborating the hypothesis of a distinct origin.

## Conclusion

In this study, we attempted to lay the foundations for the genomic and cytometric characterization of the genus *Mandevilla*. However, the road is still indisputably long. We managed to assemble the cpDNA (of great help for phylogenetic studies) and to produce a first draft of the nuclear genome, but the use of long reads-based sequencing platforms will be decisive in the future for improving the assembly (down to the chromosomal level) and for reconstructing mitochondrial DNA (mtDNA). The exclusive use of short read-based sequencing platforms (i.e., Illumina) makes the assembly of mtDNA extremely challenging and characterized by large repeated regions, nuclear and plastid-deriving sequences and several possible configurations (linear, circular, or branched) (Palumbo et al., 2021). The genomic contigs were useful to identify thousands of genes, some of which suggested the possibility that Mandevilla could produce MIAs of pharmaceutical interest, similar to many other species of the same family. Further chromatographic analyses will be crucial to elucidate if and possibly which MIAs Mandevilla is able to synthetize. The genome draft was also of great help for the development of a robust panel of SSR markers, which have recently started to be used to assist breeding programs in non-crop species, including ornamental plants. As far as cytometry is concerned, the situation turned out to be, as expected, very complex to decipher. Although the intersection of cytometry data and k-mer estimates allowed us to infer the triploidy of the reference sample used for genome sequencing, the ploidy level for the different accessions of the germplasm still remains to be defined. In fact, by comparing their FMI with the FMI values of the reference, non-integer values were obtained. This is explainable by interspecific hybridization events (partially confirmed through DNA barcoding data), which led to dysploidy phenomena, gains and losses of single chromosomes or fission and/or fusion of chromosome segments. In this context, karyological analyses coupled with genomic *in situ* hybridization (GISH) studies could represent a great deal of help in assessing the origin, divergence, relationships, and evolution of these hybrids. At the same time, even the enhancement of DNA barcoding databases could solve some uncertainties related to the possible parents of the aforementioned hybrids. In our study, assuming that *M. sanderi* was always used as maternal species, it remains to be clarified, due to lack of information, which species were used as paternal parents in the different crossing and/or backcrossing cycles of the breeding programs.

## Data availability statement

The original contributions presented in the study are publicly available. This data can be found here: NCBI, OM489306 and PRJNA802340.

## Author contributions

FP and GB: conceptualization. SD, FS, and FP: methodology. SD and FS: formal analysis. FP, SD, GG, and FS: data analysis. FP: writing—original draft preparation. FP, FS, SD, GG, GS, MG, and GB: writing—review and editing. GB: supervision and project administration. GB and MG: funding acquisition. All authors have read and agreed to the published version of the manuscript.

## Footnotes

1
https://olomouc.ueb.cas.cz/en/technology/flow-cytometry-1/reference-dna-standards

2
https://www.geneious.com
