# Supplementary material for: First genomic insights into the Mandevilla genus
Source: Front Plant Sci. 2022 Aug 16;13:983879. doi: 10.3389/fpls.2022.983879 (PMC9426028; doi:10.3389/fpls.2022.983879)
Supplement: Supplementary file 10 [file Data_Sheet_1.PDF]

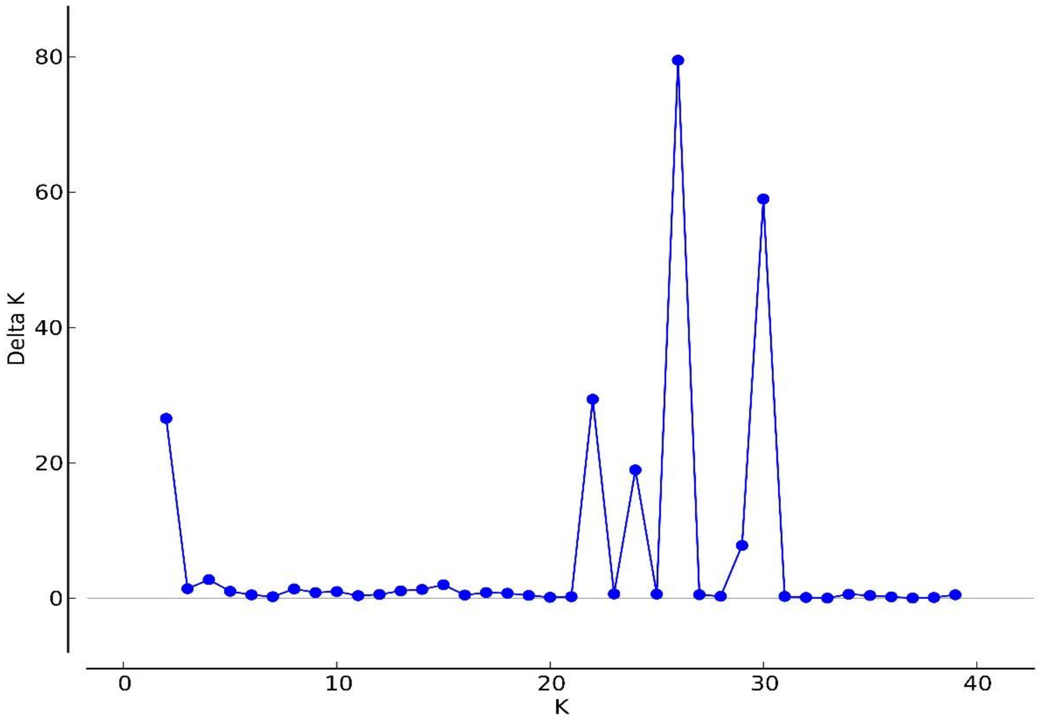

Supplementary Figure 1. Definition of the number of ancestral Mandevilla populations based on the SSR marker dataset. ΔK values are represented by the blue line, while the blue points indicate the mean LnP(D) ± SD values. Mean LnP(D) ± SD (over 10 runs) is a function of K, as L′(K) = ΔLnP(D) and mean ΔK is calculated as |L″(K)|/(SD(L(K)).
